# High-contrast and reversible scattering switching via hybrid metal-dielectric metasurfaces

**DOI:** 10.3762/bjnano.9.44

**Published:** 2018-02-06

**Authors:** Jonathan Ward, Khosro Zangeneh Kamali, Lei Xu, Guoquan Zhang, Andrey E Miroshnichenko, Mohsen Rahmani

**Affiliations:** 1 Nonlinear Physics Centre, Australian National University, Canberra ACT 2601, Australia; 2 The MOE Key Laboratory of Weak Light Nonlinear Photonics, School of Physics and TEDA Applied Physics Institute, Nankai University, Tianjin 300457, China; 3 School of Engineering and Information Technology, University of New South Wales at the Australian Defence Force Academy, Canberra, ACT 2600, Australia

**Keywords:** dielectrics, directionality, metasurfaces, Mie resonance, plasmonics, reversible tuning

## Abstract

Novel types of optical hybrid metasurfaces consist of metallic and dielectric elements are designed and proposed for controlling the interference between magnetic and electric modes of the system, in a reversible manner. By employing the thermo-optical effect of silicon and gold nanoantennas we demonstrate an active control on the excitation and interference between electric and magnetic modes, and subsequently, the Kerker condition, as a directive radiation pattern with zero backscattering, via temperature control as a versatile tool. This control allows precise tuning optical properties of the system and stimulating switchable sharp spectral Fano-like resonance. Furthermore, it is shown that by adjusting the intermediate distance between metallic and dielectric elements, opposite scattering directionalities are achievable in an arbitrary wavelength. Interestingly, this effect is shown to have a direct influence on nonlinear properties, too, where 10-fold enhancement in the intensity of third harmonic light can be obtained for this system, via heating. This hybrid metasurface can find a wide range of applications in slow light, nonlinear optics and bio-chemical sensing.

## Introduction

Metasurfaces are thin and flat surfaces that are created using subwavelength optical antennas with various optical properties patterned at interfaces [1–2], enabling control over the polarization, phase, amplitude, and dispersion of light. Metasurfaces are growing in popularity as their optical properties can be adapted to a diverse set of applications along the electromagnetic spectrum [3] including dispersion engineering [4], polarization manipulation [5–6], pulse shaping [7], sensing [8–9] and tuning [10]. The first generation of metasurfaces mostly consisted of plasmonic nanostructures [11–13], which utilize the interaction between light and metallic nanoparticles to generate surface plasmon resonances, inducing a strong electromagnetic field on the metallic surface. They benefit from a large tunability and capability to significantly enhance the near-field intensity, and have remarkable advantages in controlling optical responses [14–18].

All-dielectric, high refractive index metasurfaces are the second generation of metasurfaces [19]. Besides their CMOS compatibility and low optical losses compared with plasmonic metasurfaces, all-dielectric metasurfaces offer ability to efficiently manipulate light at the nanoscale based on the simultaneous control of electric and magnetic Mie resonances [19]. Subsequently, the capability to control electric and magnetic resonances, offers a unique platform to engineer and tune the directionality of light emission [20–21]. Kerker et al. demonstrated that light scattering can be completely suppressed in certain directions from a subwavelength scatterer when the electric and magnetic responses are of the same order (ε = μ) [22]. This causes destructive interference in the backward propagation direction and is known as the first Kerker condition. Within dielectric metasurfaces, it has been shown that an overlap of the electric and magnetic dipole resonances can generate a relatively broad spectral band Kerker condition [20,23–24].

In order to achieve high-performance compact optical devices with novel functionalities for applications in modern nanophotonics, tunability of metasurfaces is required, which has become a rapidly growing area of research. These tuning capabilities have been introduced via various techniques, such as phase-change media based antennas [25–27], the use of liquid crystals [28–30], doping [31–32], stretchable substrates [33–34], and electromechanical tuning of the resonator dimension [35], etc. Many of these techniques introduce permanent alterations to the system or the environment, which makes them irreversible, an undesirable characteristic. This allows for an active metasurface tuning mechanism that is reversible and reconfigurable.

Recently, a new technique for reversible tuning of metasurfaces has been proposed, which is based on the thermo-optical coefficient of materials [36–37]. This technique seems quite promising as it varies the temperature of the structure in an easy and reversible way. The large thermo-optical coefficient of dielectrics allows for active control of the devices optical properties by heating. This tunability can be done in a reversible and dynamic way using a parameter that is controlled externally. This tunable device then allows for flat optical components that are adjustable, like beam shapers and lenses [29]. Importantly, thermal tuning is only applicable to dielectric metasurfaces, as the low thermo-optical coefficient of most of noble metals, such as gold [38], makes plasmonic metasurfaces insensitive to variation in temperature.

Hybrid metal–dielectric nanostructures allow for combining the thermo-optical properties of both plasmonic and all-dielectric nanoantennas, simultaneously. Here, by taking advantage of different thermo-optical properties of metallic and dielectric metasurfaces, we propose a novel hybrid metasurface that provides a unique platform to tune the excitation of electric and magnetic modes and their interference in parallel. This leads to a unique capability to tune the Kerker condition in the near-IR, i.e., Kerker interference can be turned on and off in a completely reversible way. It is worth noting that this capability is not achievable with either dielectric or metallic metasurfaces alone, because electric resonance of metallic metasurfaces are essentially insensitive to heating, and magnetic and electric resonances of dielectric metasurface shift together during heating, with negligible change in the interference regime.

## Results and Discussion

**Figure 1:**
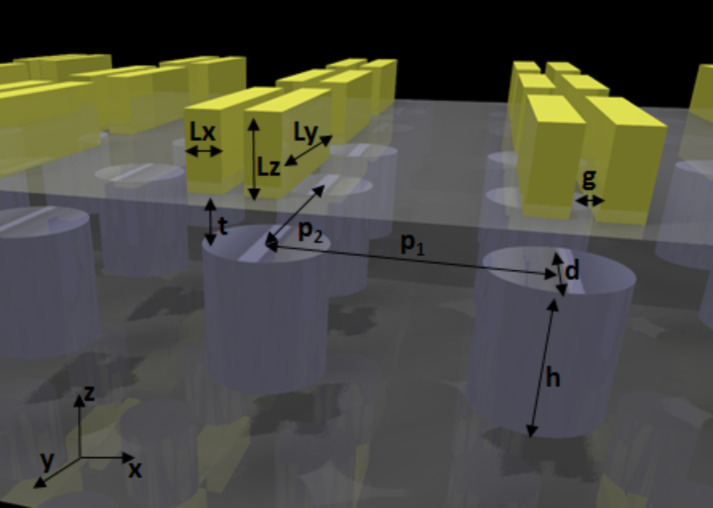
Diagram of the metasurface. The geometrical parameters are *L*_x_ = 100 nm, *L*_y_ = 600 nm, *L*_z_ = 200 nm, *t* = 200 nm, *d* = 400 nm, *h* = 400 nm, *g* = 60 nm, *p*_1_ = 850 nm, *p*_2_ = 850 nm.

**Figure 2:**
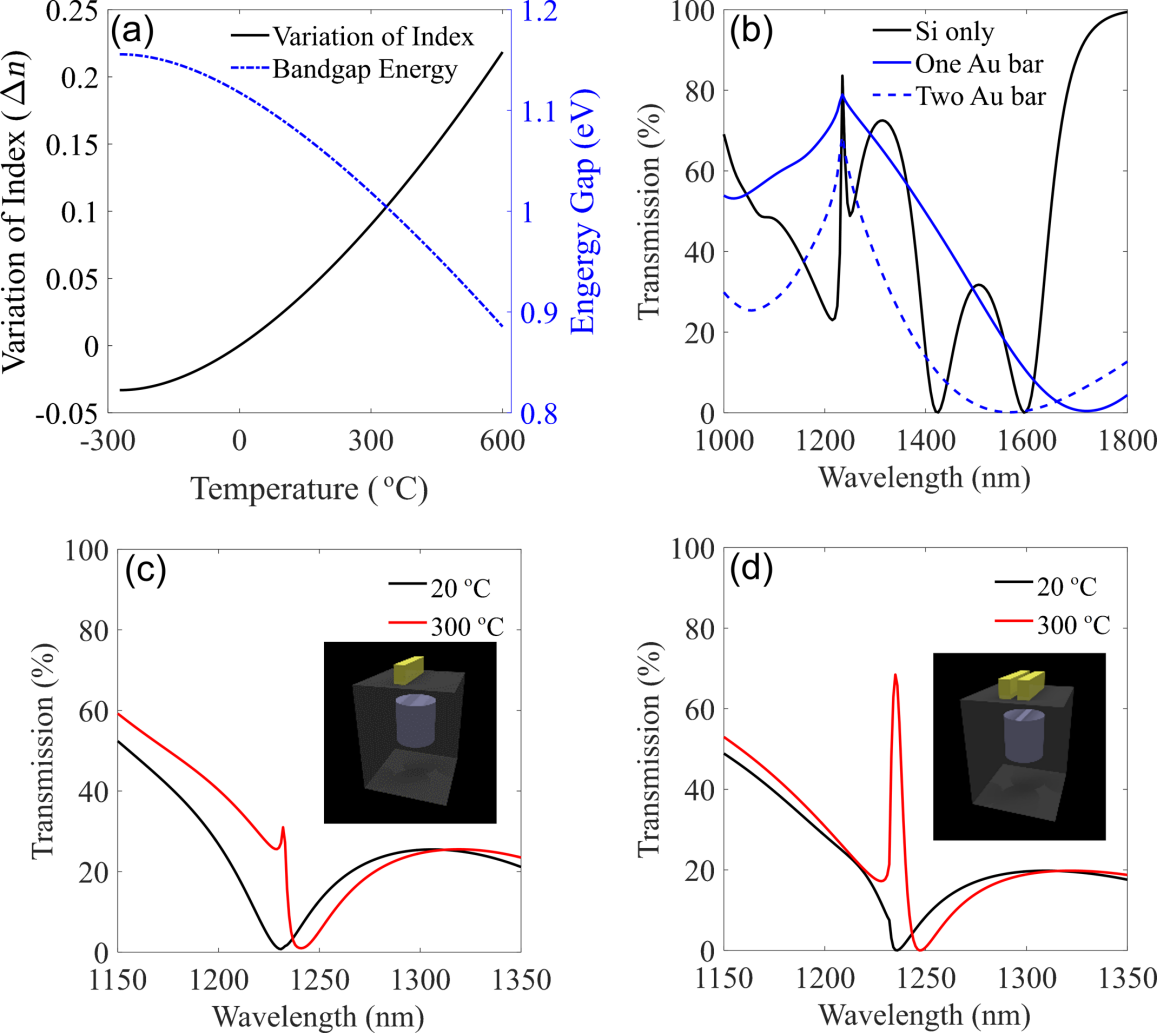
(a) Bandgap energy (blue curve) and variation of refractive index (dark curve) versus temperature change for bulk silicon [40]. (b) Transmission of silicon only (black), one gold bar (red), and two gold bars (blue) in the wavelength range 1000 to 1800 nm at room temperature. (c) The transmission of the silicon with one gold bar at room temperature (20 °C) and 300 °C, respectively. (d) The transmission of the silicon with two gold bars at room temperature (20 °C) and 300 °C, respectively.

Our proposed technique to dynamically control the interference of the magnetic and electric resonances provides higher flexibility in tailoring the scattering of light in nanostructures. By employing an additional degree of freedom; coupling between plasmonic and dielectric resonances, we are able to engineer the excitation of the resonant responses over the whole system. Our well-designed hybrid metasurface (see Figure 1) can stimulate or avoid the Kerker condition, on demand. This unique capability can help to realize directional emission from metallic and dielectric nanoantennas. Our design consists of a periodic lattice of silicon cylinders with rectangular gold bars stacked above them, separated by a thin film of SiO_2_. This metasurface is designed to stimulate a sharp interference between silicon and gold lattice resonance around a wavelength of 1235 nm (see Figure 2a). It is an arbitrary wavelength, which can be chosen by a proper design. The reasoning for the NIR range is to avoid absorption in the silicon. The reason behind choosing gold nanobars, rather than simple gold discs, is the following: the system relies on the lattice separation of silicon discs, using gold discs provides limited options for designs as the only degrees of freedom are the diameter and height of the discs. However, employing bars can address this issue by providing one more degree of freedom using the height, width and length of the bars.

The thermal dependence of the energy gap with temperature for silicon can be described by the following relation [39]:

[1]
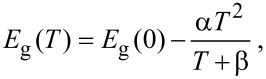


where *E*_g_(0) = 1.1557 eV is the energy gap at zero temperature, and α = 7.021 × 10^−4^ eV/K and β = 1108 K are parameters fitted from experimental data in [39]. The relation between the variation of refractive index change and the temperature for silicon is given by Tripathy as [40]

[2]
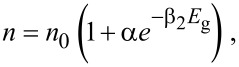


where *n*_0_ = 1.73, α = 1.9017, and β = 0.539 (eV)^−1^. Figure 2a shows the corresponding thermal dependence of variation of the energy gap and refractive index with temperature in silicon, respectively, [37]. Subsequently, we employ the thermal dependence of silicon to further explore the thermal tunability of the designed hybrid metal–dielectric nanostructures.

Figure 2b demonstrates the transmission characteristics of each element in the hybrid metasurface, including silicon disk lattice, one-gold-bar lattice, and two-gold-bar lattice, respectively. The polarization of the incident beam is along *y* direction (along the gold bar lengths). A grating resonance in the spectrum occurs around 1235 nm at the grating diffraction edge (λ_0_ = *n × D* = 1.45 × 850 nm = 1232.5 nm) due to the constructive diffractive feedback among neighbouring antennas. Therefore, as can be seen, all these components, separately, exhibit a sharp resonance at the target wavelength.

Figure 2c and 2d show the transmission spectra of the hybrid metasurfaces with one gold bar and two gold bars, respectively. As it has been recently demonstrated, heating the system causes a variation in the refractive index of silicon nanostructures which further leads to a systematic shift in the resonances of the system [37]. However, in our design, due to the employment of the thermo-optical properties of both silicon and gold simultaneously, the hybrid metasurface shows a remarkable variation in optical scattering properties. Figure 2c shows a small Fano-like resonance which can be switched on and off near the wavelength 1235 nm, via heating. Interestingly, such effect can be enhanced significantly when adding a second gold bar on the top of each silicon element (see Figure 2d). As a result, around 70% tunability of transmission can be achieved in this wavelength via heating process.

**Figure 3:**
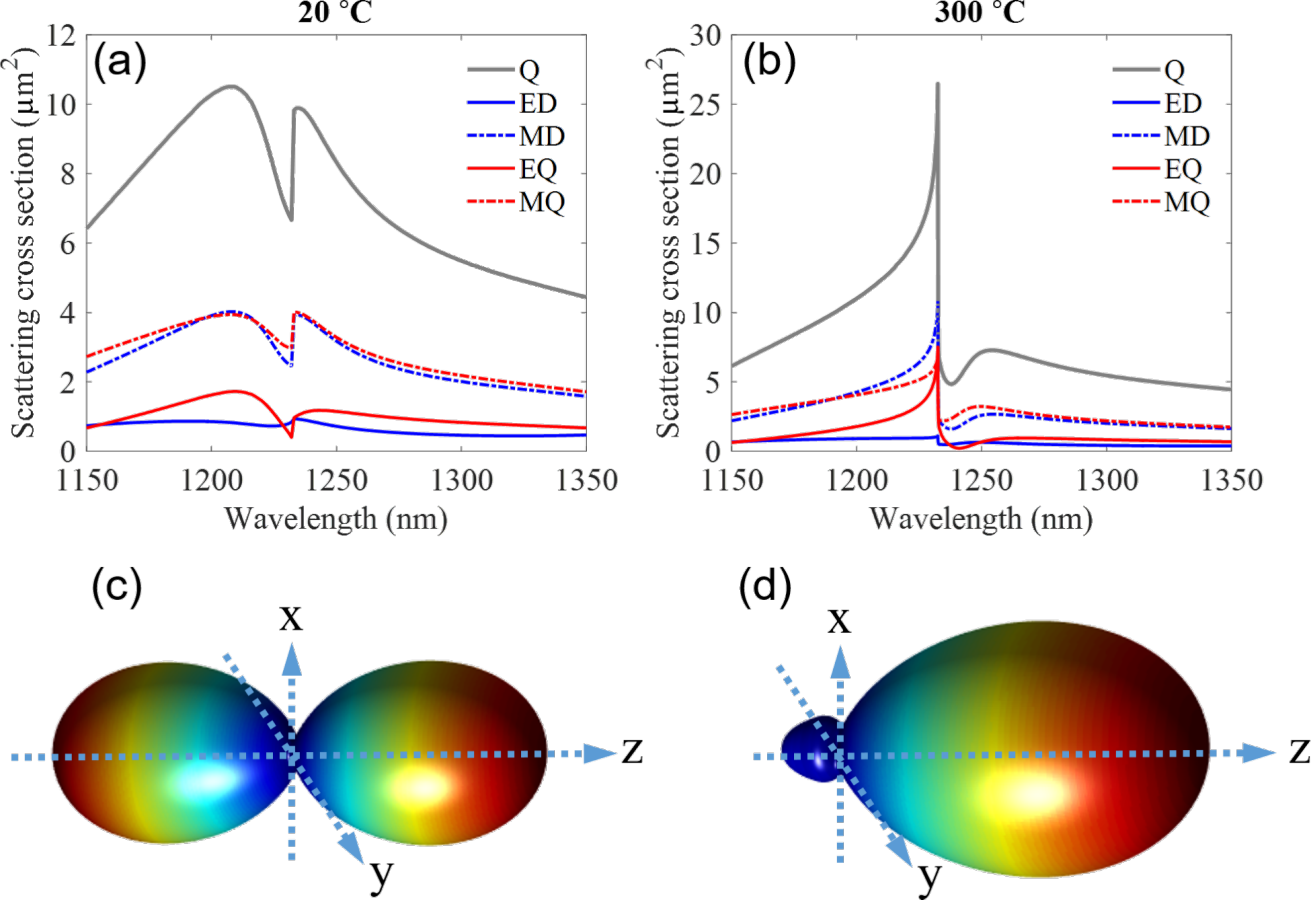
Scattering cross section (a) before and (b) after heating, including zoomed in on Kerker condition in the heated scattering cross section sample. Far-field radiation pattern (c) before and (d) after heating.

In order to get a physical insight in this phenomenon, we have studied the mode decomposition of the hybrid system consisting of a pair of Au bars [41–43]. As can be seen in Figure 3a, before heating, in the wavelength range between 1150 and 1350 nm, the total scattering (*Q*) is determined mainly by the resonant excitations from magnetic dipole (MD) and magnetic quadrupole (MQ), and a small portion of excitation from electric quadrupole (EQ) and electric dipole (ED). These optically induced responses in the metasurface can be reflected from the transmission spectra shown in Figure 2c, where the transmission is suppressed in this range (black curve).

After heating, the scattering properties can be tuned drastically and a significant Fano-like resonance appears. The corresponding multipoles excitation after heating (at 300 °C) is shown in Figure 3b. As can be seen, rather than shifting the resonances in silicon metasurfaces as demonstrated recently [37], our hybrid design allows significant changes in the resonant excitation magnitude due to the variation of the magnetic–electric coupling during heating. All modes experience a drastic change around 1235 nm, where a clear interference between electric and magnetic modes (EQ, MQ, and ED, MD) takes place. Interestingly, after heating, around 1235 nm, EQ and MQ experience a comparable magnitude that enables Kerker scattering condition and suppress the backward scattering of light. This produces the Fano-like shape in the transmission spectrum (see Figure 2c,d).

By considering the response of a single unit cell, based on the excitation of the electric and magnetic multipole moments inside it, we further visualize the associated far field pattern from such a single element. Taking the working wavelength being 1235 nm, as an example, Figure 3c and Figure 3d show the results before and after heating, respectively. Before heating, the forward and backward scattering are almost the same, which corresponds to a low transmission from the metasurface based on the scattering properties of resonant nanoparticles and metasurfaces [44–45]. However, after heating the sample to 300 °C, most of the scattered field is in the forward direction, and the backward scattering is suppressed. High scattering directionality is achieved based on the Kerker scattering condition. Similar to Huygens’ metasurfaces [23], a high transmission can be observed in the spectra of the hybrid metasurface (see Figure 2c).

**Figure 4:**
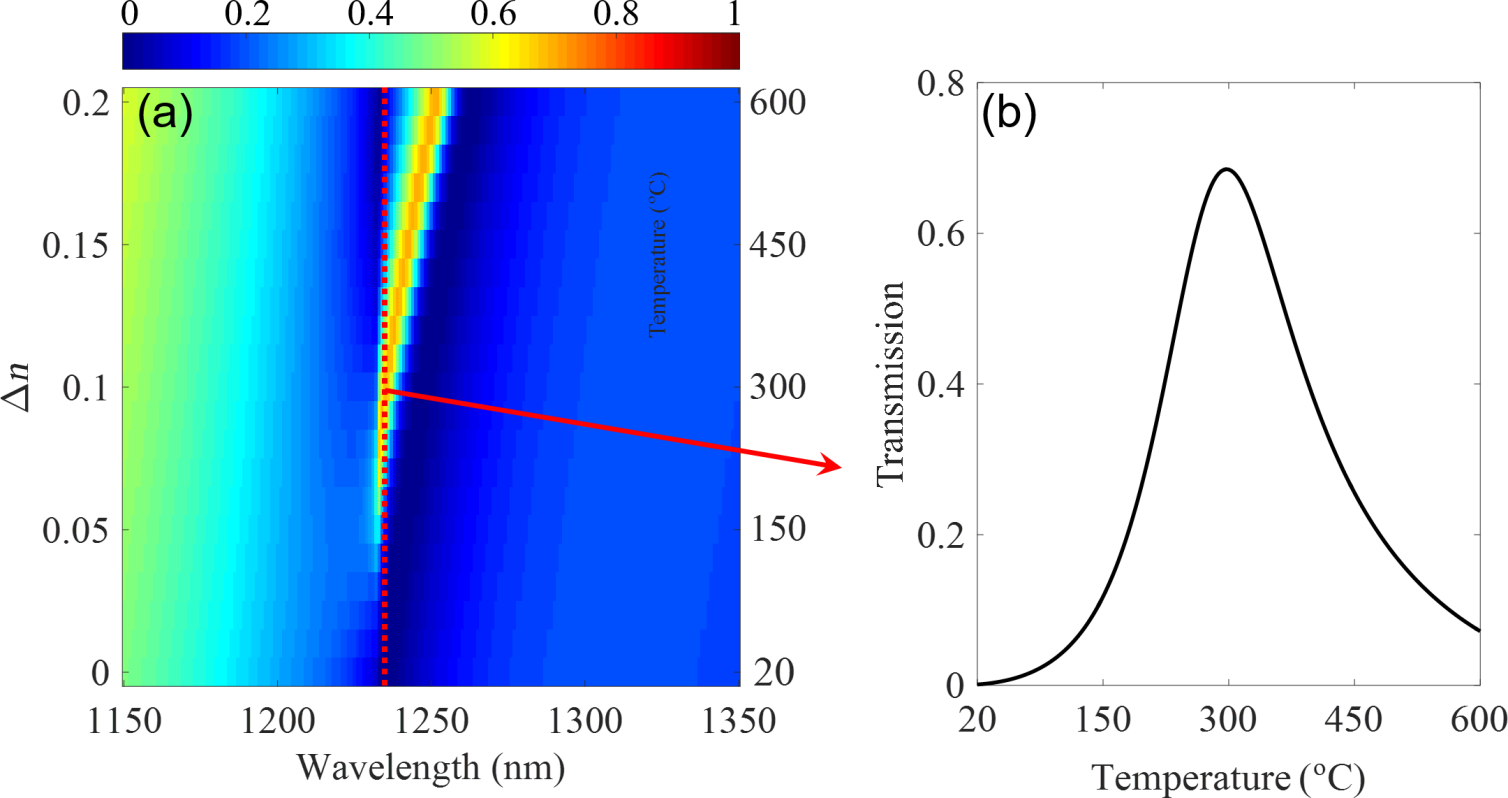
(a) The transmission spectrum during the heating process. (b) The transmission at 1235 nm during the heating process.

Interestingly, heating not only provides a reversible change in the optical properties, but also a dynamical one. Figure 4 shows the transmission spectrum during the heating process. As can be seen, there is a further redshift with increasing temperature, providing unique control on the light scattering from such hybrid metasurface. Take λ = 1235 nm as an example, Figure 4b shows the related transmission with increasing temperature during the heating process. In this case, the system exhibits zero transmission at room temperature, and after heating the sample to 300 °C, high transmission from the metasurface can be achieved. However, with further heating the sample to 600 °C, the transmission at such wavelength can be suppressed again through the same system.

**Figure 5:**
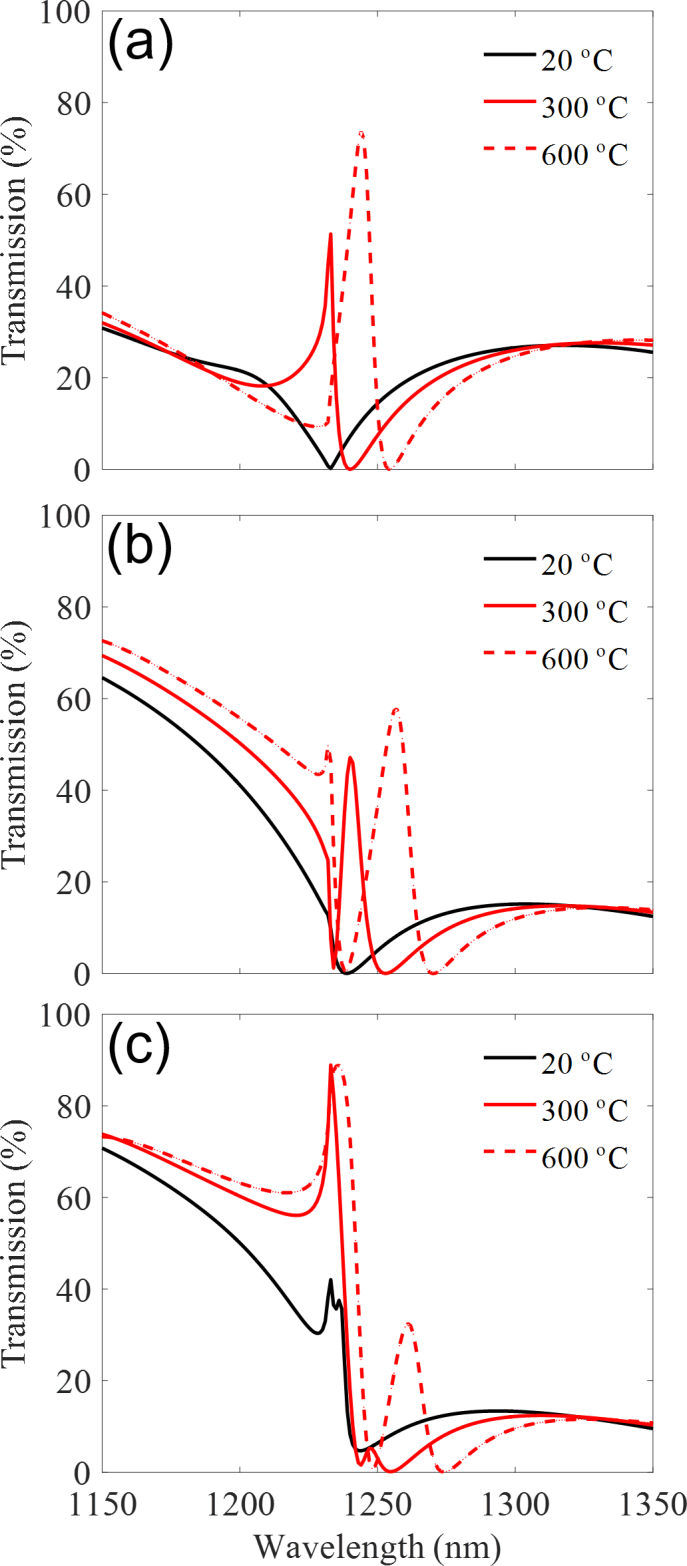
The transmission spectrum for three different SiO_2_ thicknesses (a) *t* = 150 nm, (b) *t* = 250 nm and (c) *t* = 300 nm.

Both plasmonic and dielectric modes exhibit intense near-field distributions [8–9,46]. It is well-known that the near-field associated with plasmonic and dielectric resonances of the individual particles extends some distance away from it [47–48]. These characteristics make the modes very sensitive to the environment [8–9,46]. Therefore, the interference between adjacent resonators within our hybrid system strongly depends on the environment near the nanostructures, such as the volume and refractive index. By employing this sensitivity, we further control the optical response of the metasurface by engineering the interactions between plasmonic and dielectric resonances through tuning the geometry of the intermediate SiO_2_ layer. By altering the design slightly, one can make the metasurface behave differently in response to heat. Figure 5 shows the effect of the intermediate SiO_2_ layer between silicon and gold structures. It demonstrates the importance of this layer in obtaining different and even reversible behaviour using the exact same silicon and gold nanostructures. Figure 5a demonstrate the case with *t* = 150 nm, where the heating from 20 °C to 600 °C causes only an enhanced transmission with a slight shift. However for the case of *t* = 250 nm (Figure 5b), such a heating gradient, can cause a clear fluctuation, where the system experiences an increase (solid red) and then a significant decrease (dashed red) at 1235 nm. Figure 5c illustrates an even larger disparity between the two elements where the minimum no longer exist and the resonance only grows when heated.

**Figure 6:**
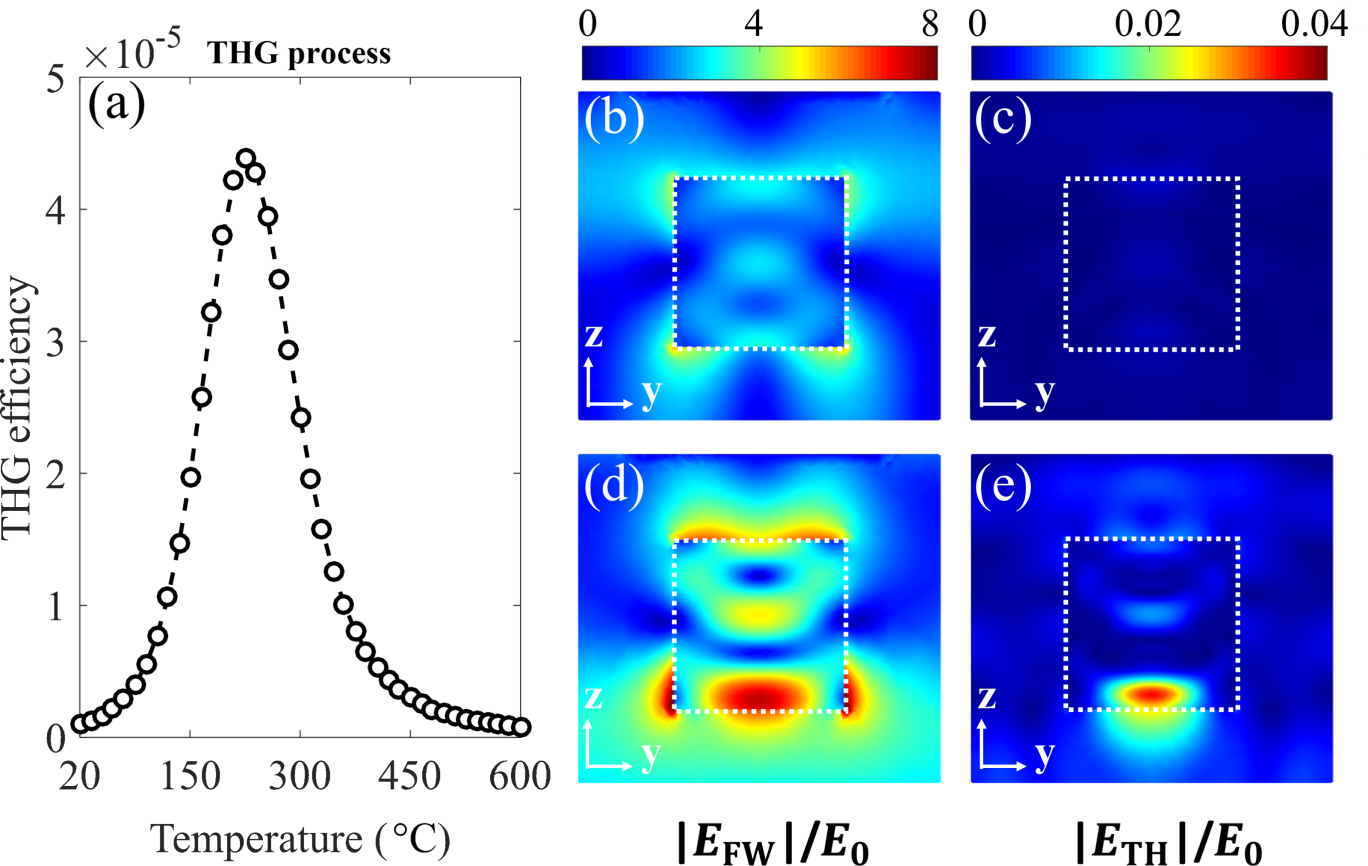
(a) Third harmonic generation efficiency (normalized by the incident pump for each unit cell) during the heating process pumped at the wavelength of 1235 nm. (b) and (c) show the electric near-field distributions at fundamental (left) and harmonic (right) wavelengths in the silicon disk at room temperature, respectively. (d) and (e) show the related calculations after heating the sample at 275 °C.

By utilizing such well-designed Fano-like resonance, further study was done on its effects on the nonlinear process, focusing on the third harmonic generation. Figure 6 shows the third harmonic conversion efficiency and the related electric near-field distributions in the silicon disk for a plane wave with λ_FW_ = 1235 nm at *I*_0_ = 1 GW/cm^2^. As can be seen from Figure 6a, the efficiency of third harmonic generation can be enhanced by one order of magnitude due to the emergence of the Fano-like resonance during the heating process, from ≈10^−6^ to more than 4 × 10^−5^. The calculated electric near-field distributions at the fundamental and harmonic wavelengths are depicted in Figure 6b and 6c for the sample at room temperature and Figure 6d and 6e after heating the sample to about 275 °C, respectively. With the emergence of the Fano-like resonance during the heating process, strong field localization and enhancement inside the silicon disk occurs as a result of the excitation and interferences between optically-induced electric and magnetic multipoles (see Figure 3a and 3b). The enhancement of the electric field further stimulates the nonlinear response, as can be clearly seen from the comparison between the electric near-field distributions at the harmonic wavelengths before and after heating the sample (Figure 6c and 6e). It is worth noting that while gold bars show a significant effect on the linear properties of the system after heating, the THG from gold bar is negligibly small compared to THG from silicon disk. It is because the light of the hybrid resonance is generally confined inside the silicon disk rather than the gold bars.

## Conclusion

We have designed a hybrid metal–dielectric metasurface, composed of silicon disk and gold bar lattices, with a reversible switching capability of the Kerker scattering condition. The tunability is achieved by the active control on the excitation and interference between the electric and magnetic resonances through heating the metasurface. It is shown that the Kerker condition, based on high order multipoles (EQ and MQ), can be easily switched on and off by simply heating the system. Furthermore, through adjustment to the distance between the metallic and dielectric elements, scattering directionality can be switched at arbitrary wavelengths. We further investigated the THG process in such hybrid meta-dielectric metasurface, and have achieved multi-fold enhancement of THG based on the well-designed hybrid resonance during the heating process. Our hybrid metasurface provides much more flexibility for the control of light scattering, which serves as an important step towards tunable flat optics.

## Methods

Here, we study the transmission properties of the nanostructures using rigorous coupled-wave analysis (RCWA) method [49–50] which has been widely used for modelling periodic optical structures due to its fast converging and accurate far-field calculations. We characterize the optical properties of the sample during the heating process based on the refractive index variations of the material at different temperatures, which has been measured experimentally [37].

The nonlinear process of our sample is emulated using the finite element method solver in COMSOL Multiphysics in the frequency domain. We assume an undepleted pump approximation and simulate the linear process firstly, and then obtain the nonlinear polarization inside the sample and employ it as a source for the electromagnetic simulation at the harmonic wavelength [43].

## References

[1] Yu N, Capasso F (2014). Nat Mater.

[2] Zheludev N I, Kivshar Y S (2012). Nat Mater.

[3] Mousavi S H, Kholmanov I, Alici K B, Purtseladze D, Arju N, Tatar K, Fozdar D Y, Suk J W, Hao Y, Khanikaev A B (2013). Nano Lett.

[4] Guo Y, Wang Y, Pu M, Zhao Z, Wu X, Ma X, Wang C, Yan L, Luo X (2015). Sci Rep.

[5] Chen X, Huang L, Mühlenbernd H, Li G, Bai B, Tan Q, Jin G, Qiu C-W, Zhang S, Zentgraf T (2012). Nat Commun.

[6] Aieta F, Genevet P, Kats M A, Yu N, Blanchard R, Gaburro Z, Capasso F (2012). Nano Lett.

[7] Walther B, Helgert C, Rockstuhl C, Setzpfandt F, Eilenberger F, Kley E-B, Lederer F, Tünnermann A, Pertsch T (2012). Adv Mater.

[8] Bontempi N, Chong K E, Orton H W, Staude I, Choi D-Y, Alessandri I, Kivshar Y S, Neshev D N (2017). Nanoscale.

[9] Yavas O, Svedendahl M, Dobosz P, Sanz V, Quidant R (2017). Nano Lett.

[10] Kildishev A V, Boltasseva A, Shalaev V M (2013). Science.

[11] Meinzer N, Barnes W L, Hooper I R (2014). Nat Photonics.

[12] Geraci G, Hopkins B, Miroshnichenko A E, Erkihun B, Neshev D N, Kivshar Y S, Maier S A, Rahmani M (2016). Nanoscale.

[13] Gennaro S D, Rahmani M, Giannini V, Aouani H, Sidiropoulos T P H, Navarro-Cia M, Maier S A, Oulton R F (2016). Nano Lett.

[14] Pors A, Nielsen M G, Bozhevolnyi S I (2015). Optica.

[15] Keren-Zur S, Avayu O, Michaeli L, Ellenbogen T (2016). ACS Photonics.

[16] Wu P C, Tsai W-Y, Chen W T, Huang Y-W, Chen T-Y, Chen J-W, Liao C Y, Chu C H, Sun G, Tsai D P (2017). Nano Lett.

[17] Lee J, Tymchenko M, Argyropoulos C, Chen P-Y, Lu F, Demmerle F, Boehm G, Amann M-C, Alù A, Belkin M A (2014). Nature.

[18] Almeida E, Shalem G, Prior Y (2016). Nat Commun.

[19] Kuznetsov A I, Miroshnichenko A E, Brongersma M L, Kivshar Y S, Luk’yanchuk B (2016). Science.

[20] Liu W, Kivshar Y S (2017). Philos Trans R Soc, A.

[21] Staude I, Miroshnichenko A E, Decker M, Fofang N T, Liu S, Gonzales E, Dominguez J, Luk T S, Neshev D N, Brener I (2013). ACS Nano.

[22] Kerker M, Wang D-S, Giles C L (1983). J Opt Soc Am.

[23] Decker M, Staude I, Falkner M, Dominguez J, Neshev D N, Brener I, Pertsch T, Kivshar Y S (2015). Adv Opt Mater.

[24] Kruk S, Hopkins B, Kravchenko I I, Miroshnichenko A, Neshev D N, Kivshar Y S (2016). APL Photonics.

[25] Michel A-K U, Chigrin D N, Maß T W W, Schönauer K, Salinga M, Wuttig M, Taubner T (2013). Nano Lett.

[26] Wang Q, Rogers E T F, Gholipour B, Wang C-M, Yuan G, Teng J, Zheludev N I (2016). Nat Photonics.

[27] Li P, Yang X, Maß T W W, Hanss J, Lewin M, Michel A-K U, Wuttig M, Taubner T (2016). Nat Mater.

[28] Minovich A, Farnell J, Neshev D N, McKerracher I, Karouta F, Tian J, Powell D A, Shadrivov I V, Hoe Tan H, Jagadish C (2012). Appl Phys Lett.

[29] Sautter J, Staude I, Decker M, Rusak E, Neshev D N, Brener I, Kivshar Y S (2015). ACS Nano.

[30] Komar A, Fang Z, Bohn J, Sautter J, Decker M, Miroshnichenko A, Pertsch T, Brener I, Kivshar Y S, Staude I (2017). Appl Phys Lett.

[31] Jun Y C, Brener I (2012). J Opt (Bristol, U K).

[32] Lewi T, Iyer P P, Butakov N A, Mikhailovsky A A, Schuller J A (2015). Nano Lett.

[33] Kamali S M, Arbabi A, Arbabi E, Horie Y, Faraon A (2016). Nat Commun.

[34] Gutruf P, Zou C, Withayachumnankul W, Bhaskaran M, Sriram S, Fumeaux C (2016). ACS Nano.

[35] Liu A Q, Zhu W M, Tsai D P, Zheludev N I (2012). J Opt (Bristol, U K).

[36] Lewi T, Evans H A, Butakov N A, Schuller J A (2017). Nano Lett.

[37] Rahmani M, Xu L, Miroshnichenko A E, Komar A, Camacho-Morales R, Chen H, Zárate Y, Kruk S, Zhang G, Neshev D N (2017). Adv Funct Mater.

[38] Palpant B, Rashidi-Huyeh M, Gallas B, Chenot S, Fisson S (2007). Appl Phys Lett.

[39] Varshni Y P (1967). Physica (Amsterdam).

[40] Tripathy S K (2015). Opt Mater.

[41] Bakker R M, Permyakov D, Yu Y F, Markovich D, Paniagua-Domínguez R, Gonzaga L, Samusev A, Kivshar Y, Luk'yanchuk B, Kuznetsov A I (2015). Nano Lett.

[42] Evlyukhin A B, Fischer T, Reinhardt C, Chichkov B N (2016). Phys Rev B: Condens Matter Mater Phys.

[43] Wang L, Kruk S, Xu L, Rahmani M, Smirnova D, Solntsev A, Kravchenko I, Neshev D, Kivshar Y (2017). Nanoscale.

[44] Bohren C F, Huffman D R (1983). Absorption and Scattering of Light by Small Particles.

[45] Chong K E, Staude I, James A, Dominguez J, Liu S, Campione S, Subramania G S, Luk T S, Decker M, Neshev D N (2015). Nano Lett.

[46] Yu B, Woo J, Kong M, O’Carroll D M (2015). Nanoscale.

[47] Rahmani M, Luk'yanchuk B, Hong M (2013). Laser Photonics Rev.

[48] Nielsen M P, Lafone L, Rakovich A, Sidiropoulos T P H, Rahmani M, Maier S A, Oulton R F (2016). Nano Lett.

[49] Hugonin J P, Lalanne P (2005). Reticolo software for grating analysis.

[50] Moharam M G, Grann E B, Pommet D A, Gaylord T K (1995). J Opt Soc Am A.

